# Joint Biofortification of Plants with Selenium and Iodine: New Field of Discoveries

**DOI:** 10.3390/plants10071352

**Published:** 2021-07-02

**Authors:** Nadezhda Golubkina, Anastasia Moldovan, Helene Kekina, Victor Kharchenko, Agnieszka Sekara, Viliana Vasileva, Liubov Skrypnik, Alessio Tallarita, Gianluca Caruso

**Affiliations:** 1 Laboratory Analytical Department, Federal Scientific Center of Vegetable Production, Moscow 143072, Russia; nastiamoldovan@mail.ru (A.M.); kharchenkoviktor777@gmail.com (V.K.); 2 Medical Academy of Post Graduate Education, Moscow 123995, Russia; lena.kekina@mail.ru; 3 Department of Horticulture, Faculty of Biotechnology and Horticulture, University of Agriculture, 31-120 Krakow, Poland; agnieszka.sekara@urk.edu.pl; 4 Institute of Forage Crops, 89 General Vladimir Vazov Str, 5802 Pleven, Bulgaria; viliana.vasileva@gmail.com; 5 Institute of Living Systems, Immanuel Kant Baltic Federal University, Kaliningrad 236040, Russia; LSkrypnik@kantiana.ru; 6 Department of Agricultural Sciences, University of Naples Federico II, 80055 Portici, Naples, Italy; lexvincentall@gmail.com (A.T.); gcaruso@unina.it (G.C.)

**Keywords:** selenium, iodine, agricultural crops, sprouts, AMF

## Abstract

The essentiality of selenium (Se) and iodine (I) to human beings and the widespread areas of selenium and iodine deficiency determine the high significance of functional food production with high levels of these elements. In this respect, joint biofortification of agricultural crops with Se and I is especially attractive. Nevertheless, in practice this topic has raised many problems connected with the possible utilization of many Se and I chemical forms, different doses and biofortification methods, and the existence of wide species and varietal differences. The limited reports relevant to this subject and the multiplicity of unsolved questions urge the need for an adequate evaluation of the results obtained up-to-date, useful for developing further future investigations. The present review discusses the outcome of joint plant Se–I biofortification, as well as factors affecting Se and I accumulation in plants, paying special attention to unsolved issues. A particular focus has been given to the prospects of herb sprouts production enriched with Se and I, as well as the interactions between the latter microelements and arbuscular-mycorrhizal fungi (AMF).

## 1. Introduction

An adequate consumption of essential elements, such as Fe, Zn, Se, and I is one of the most important factors for maintaining good human health conditions. Among the above-mentioned elements, Se and I draw special attention due to the close relationship between their metabolism in mammals, where the fundamental function of Se is its presence in the active center of triiodothyronine deiodinases, involved in the biosynthesis of thyroid hormones [1,2,3], in addition to its participation in other enzymes involved in antioxidant defense: glutathione peroxidases, thioredoxin reductase, selenophosphate synthetase and some special proteins (Sel P, W, etc.) [4]. This fact entails the need to optimize the joint Se–I consumption levels associated with food products for guaranteeing thyroid and reproductive functions, improving immunity, and protecting against viral, cardiovascular diseases and cancer [1,4]. The biofortification of agricultural crops with both elements is especially attractive, as the biofortified plants are able to increase antioxidant defense of humans in addition to the presence of other natural antioxidants such as vitamins, polyphenols, carotenoids, etc. [5,6]. Moreover, plants can convert highly toxic inorganic soil Se forms into well-absorbed organic derivatives with more powerful protective effect [7]. Considering the widespread Se and I deficiency within the populations of many countries worldwide [8], the importance of the joint plant Se–I biofortification is apparent [9].

Based on the literature reports indicating a high variability of the results and the complexity of this topic, the present review is aimed both to provide the current status and to trigger future investigations relevant to the joint Se–I biofortification of agricultural crops, so as to develop and implement the technology of functional food production enabling to decrease, at least partially, the ecological risks connected with the deficiency of these two microelements. 

## 2. Consumption Levels

The main sources of Se and I for plants are soil and precipitation, with the latter providing a significant transfer of the two elements from the surface of sea and oceans [7]. Phytoplankton activity is one the most important sources of these elements entering the atmosphere predominantly as volatile methylated forms [7]. Though neither Se nor I are essential to most known plants, they are vital for several sea microalgae species [10]. Although I compounds are transferred with aerosols to remarkable distances from the seashore, the inland regions in the world always suffer from lack of I causing global problems of I deficiency [11], which along with the wide distribution of Se-deficient soils, results in serious ecological risks.

In this respect, the agrochemical biofortification of plants with Se and I is especially attractive, since it provides a chance to produce functional food products with a significant content of well-assimilated forms of trace elements and other natural antioxidants. Furthermore, most of agricultural crops belong to non-accumulators of these elements and therefore are highly sensitive to toxic Se–I levels, thus providing the so-called ‘buffer effect’ preventing human toxicities in case of overdosing during biofortification [12]. In general, the inhibition of plant growth due to toxic levels of Se and I is a well-known phenomenon occurring in many agricultural crops [12,13]. The nutritional role of Se and I, their participation in plant antioxidant defense as well as in the improvement of sugar levels, the risk of their toxicity, have been appropriately described in previous reviews [12,13]. Some examples of Se and I beneficial effects, reported in recent papers, are presented in Table 1. The data indicate the attractiveness of Se and I application to different agricultural crops, including vegetables, fruit trees and spices, and show the possibility to increase not only Se and I content, but also antioxidant levels. Furthermore, the evaluation of consumers’ willingness to purchase Se and I biofortified apples indicates the preference of biofortified fruits compared to Se and I food supplements [14]. 

In general, Se content in most agricultural crops, known to be non-accumulators of Se, may be increased up to 0.2–3000 mg kg^−1^ d.w. without growth inhibition, depending on the Se form and dose [12], as it also happens for I supplementation [11]. Considering the 80–90% water content in most vegetables and the daily Se and I requirements (Table 2), such biofortification values are capable to fulfill up to 100% of the Se and I adequate consumption levels.

## 3. Selenium and Iodine Biochemical Characteristics

Se and I are not essential for plants but, thanks to their antioxidant properties, they are able to protect plants from different forms of oxidant stress and at certain concentrations may serve as growth stimulators [5,13]. On the other hand, according to Shelford’s law of tolerance [28], the impact of any environmental factor on a living organism has its own optimum, outside of which, with an excess or deficiency of the influencing component, the inhibition of growth and development will be manifested.

In plants, the aforementioned threshold referred to Se and I is species- and variety-dependent, which determines significant differences in the enrichment efficiency with these microelements [16,21,25,29,30].

**Figure 1:**
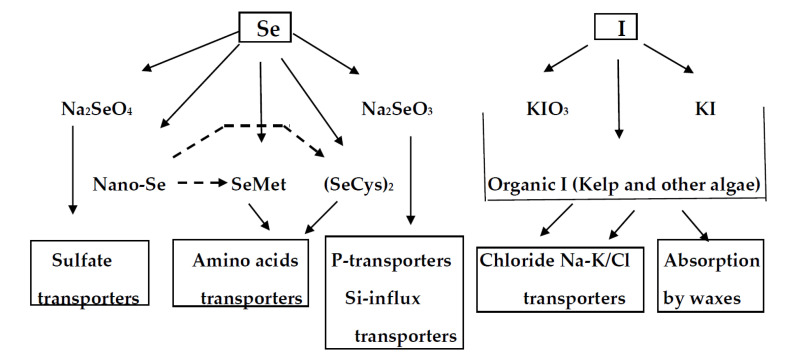
The main chemical forms and sources of Se and I used for plant biofortification and mechanisms of their absorption.

As a sulfur analog, Se has several forms with different valences: selenates (+6), selenites (+4), organic derivatives (–2; for instance, selenium containing amino acids) and elemental selenium (0; for instance, selenium nanoparticles). The known chemical forms of I are iodides (–1), iodates (+5) and derivatives of aromatic amino acids (–1) (Figure 1). 

The assimilation of both Se and I is achieved with the participation of appropriate protein-transporters (Figure 1): sulfate transporters for selenates, amino acid transporters for Se organic forms, and phosphorous and silicon transporters for selenites [31]. The accumulation of Se nanoparticles is inversely correlated with the particle size. Moreover, in plants, Se nanoparticles (NP) are freely oxidized to Se^+4^ and later transformed to selenocystine (SeCys)_2_, Se-methyl-selenocysteine (MeSeCys), and selenomethionine (SeMet) [32]. As far as iodine compounds are concerned, their assimilation is achieved via chloride transporters, Na-K/Cl transporters (I_2_, CH_3_I, KI, KIO_3_), and apparently via amino acid transporters for organic I forms [31]. Another way of I assimilation is the absorption of volatile iodine derivatives by vegetable waxes [33].

All these forms of Se and I are used for producing vegetables fortified either with Se or I [11,31].

Under joint application of the two elements, the range of their chemical forms used is limited to selenates (+6), selenites (+4), and also iodates (+5) and iodides (–1) [9].

The up-to-date results relevant to the joint plant biofortification with Se and I are presented in Table 3.

Attempts to quickly solve the deficiency problem of a whole set of trace elements, such as Se, I, Fe, Zn, in the conditions of different countries worldwide, were achieved in cereals (10 wheat cultivars and 7 rice cultivars) in 2019 and 2020, using foliar application of elements cocktail. These works revealed the efficiency of such biofortifications, but lack of significant effect on wheat yield and just a slight increase of rice yield [29,30]. In those conditions, the multiplicity of microelements used did not allow the evaluation of their interaction, particularly between Se and I. The only exception was a decrease of I accumulation in wheat under microelements cocktail utilization, compared to singly potassium iodide supply [29]. However, interesting prospects of agrochemical enrichment of cereals with several elements, recorded in these studies, have not solved the issue relevant to both choosing the optimal enrichment conditions to increase the yield and to identify in deeper detail the relationships between the elements applied. In this respect, the utilization of only two components, Se and I, provides greater opportunities for the solution of the abovementioned problems, though the approach remains still rather complex due to the wide range of either chemical forms of these elements or technological methods of supplementation. The main approaches to this study were: (1) seed germination in presence of Se and I salts; (2) supply of Se and I to soil; (3) hydroponics; and (4) foliar application of these microelements (Table 3).

## 4. Different Technological Approaches

### 4.1. Sprouts and Microgreens

The success of Se enrichment of various agricultural crop seedlings [50,51] and the popularity of this functional food among the consumers stimulated the investigations of joint Se and I application to sprouts. The data presented in Table 3 indicate that at similar Se and I dose the biofortification effect caused by these elements on sprout biomass is closely species-dependent. Indeed, the highest biomass increase was recorded in buckwheat sprouts, where joint Se–I application provided 50–70% higher microgreens biomass compared to samples singly treated with Se and I [34]. In similar conditions, the joint application of Se and I caused a slight inhibition of pea sprout development compared to plants fortified separately with Se and I [35]. Though the latter authors proposed to use the soaked seeds for planting, such an approach may cause significant risks for birds due to Se toxicity. Pumpkin sprout treatment with combined sodium selenate and potassium iodide at the same concentrations improved seed germination but did not affect yield [36].

Chervil seeds, highly sensitive to high concentrations of Se, increased seedling root length in conditions of sodium selenate and potassium iodate application at concentrations of 5 µM (that corresponds to about 1 mg sodium selenate per L), while both joint and separate application of potassium iodide and sodium selenate resulted in growth inhibition [37]. It is significant that under these conditions the total antioxidant activity (TAA) and polyphenol content (TP) in seedlings increased only under KIO_3_ and KIO_3_ + Na_2_SeO_4_ supply, indicating the development of oxidant stress (Table 4).

In the aforementioned conditions, the Se–I interaction was also highly variable under the joint application of the two elements. Indeed, in pea and chervil seedlings there was no relationship between the two elements (Table 3 and Table 4). Joint application of Se and I to buckwheat seedlings increased Se and decreased I content by twice compared to seedling supplied singly with Se and I [34]. In pumpkin seedlings a synergism between Se and I was recorded, while mature plants under joint Se–I supply showed the increased levels of Se but not of I, compared to separate supplementation of the two elements [36]. The results indicate that Se–I interaction is highly species-dependent, suggesting the remarkable genetic influence on this phenomenon. However, the lack of a general pattern indicates the need for further investigations for disclosing the factors raising the plant behavior differences in a wide range of species, especially those mainly used for production of seedlings and microgreens (broccoli, radish, cauliflower, cabbage, kale, kohlrabi, mustard, mizuna, cress, etc.) [52,53]. In addition, according to Galieni et al. [53], a priority target might be represented by species of Brassicacea, Asteracea, Amaranthaceae, Boraginacea, Convolvulaceae, Malvaceae, Poaceae, Lamiaceae, Leguminosae, Onagraceae and Portulacaceae families. In this respect, special attention should be paid to the effect of Se–I biofortification on antioxidant characteristics of sprouts and microgreens, as the latter are known for their extremely high levels of antioxidants due to the stimulating effect of germination on secondary metabolites biosynthesis and phytochemical content increase [54]. Such an approach may open new horizons in production of functional food, highly valuable both for maintaining good human health conditions and preventing various diseases. At present, these opportunities of joint Se–I sprout biofortification are still to be achieved. Furthermore, the information gained from such investigations may give additional benefits for evaluating the species differences in plant tolerance to different forms of Se and I, as well as the patterns of Se–I relationship in plants.

### 4.2. Hydroponics

Hydroponics or soilless is another technological approach providing strict control of crop growing conditions and minimizing the effect of environmental factors. Four studies of joint Se–I biofortification in soilless conditions [38,39,40,41] demonstrated the stimulating effect of salicylic acid on I accumulation. Furthermore, in conditions of joint Se–I hydroponics application, the activation of Se organic forms biosynthesis (SeMet, SeCys) reportedly takes place [38,41]. Though phytohormones are known to participate in plant Se accumulation [55,56], induce enrichment of chlorella with omega-3 fatty acids [57], take part to plant protection against heavy metals uptake [40,58], the investigations of Smolen et al. [38,39,40,41] did not reveal any significant interaction between salicylic acid and Se.

Experiments with *Arabidopsis thaliana* revealed that micromolar concentrations of I are beneficial for biomass accumulation and lead to early flowering, regulating the expression of several genes mostly involved in the plant defense response, and may be incorporated into proteins both of shoot, participating in the photosynthesis, and of roots associated with some peroxidase activities [13]. 

Unfortunately, no direct data on phytohormones participation in plant I accumulation are available, except the Smolen experiments with salicylic acid [38,39] (Table 3). Joint biofortification of lettuce with Se and I under salicylic acid supply resulted in the enhancement of leaf sugar content and changes in phosphorus and manganese accumulation in roots [38], and like in other technological approaches, great varietal differences in Se and I accumulation were reported [39]. The latter authors, in experiments with potato, revealed an increase in tuber N, Na and K content and a decrease of Mn and Zn.

### 4.3. Selenium/Iodine Soil Application

Great success in the utilization of Se-containing fertilizers in Finland [59] for optimizing human Se status led to attempts to incorporate Se and I in plants via soil application. The reason for the scant data regarding soil Se–I supply for joint biofortification of agricultural crops is connected with the complexity of environmental factors affecting the enrichment process, such as soil characteristics and microbial community, and intensive I absorption by soil components [60,61]. Appropriate investigations on soil Se–I biofortification developed by Smolen on lettuce and carrot, may be considered fundamental in this respect. These works revealed a higher efficiency of KI and Na_2_SeO_4_ supply compared to KIO_3_ and Na_2_SeO_3_ use. Despite a significant increase in SeMet and SeCys content in lettuce leaves due to joint Se–I application, the authors demonstrated a decrease in Se and I concentration in joint experiments compared to the separate application of the two elements [43]. Furthermore, joint biofortification of carrot with Se and I resulted in a decrease of sugar, carotenoids and dry matter in roots, without any significant effect on root yield and lack of Se–I interaction. The abovementioned results indicated the need of utilizing other technological approaches for producing vegetables enriched with Se and I.

### 4.4. Foliar Biofortification

Among different methods of joint Se–I biofortification, foliar application of the two elements is maybe the most interesting, although the technology of such enrichment raises some risks due to Se toxicity. As shown in Table 3, five out of six examples of Se–I supply demonstrated significant Se–I interaction. Interestingly, while pea sprouts decreased their biomass due to joint Se–I supplementation, foliar application of the same doses improved the biomass of mature pea plants. Supposedly, the application of lower Se–I concentrations should be used for sprouts compared to mature plants. Indeed, the same phenomenon was noted for Se biofortification of chervil: 25 mg Se L^−1^ concentration was optimal for mature plants [37,62] and toxic for chervil sprouts and only 1 mg Se L^−1^ did not affect the sprout biomass.

Notably, foliar application of the two elements resulted in significant Se–I interaction compared to the hydroponic conditions which did not cause significant effects of the joint Se–I supply, and only 50% of the investigations relevant to sprout Se–I biofortification revealed the existence of Se–I relationships. Foliar biofortification technology demonstrated that Se–I interaction is highly species-dependent. Indeed, while Se–I joint foliar biofortification of chicory resulted in the increase of selenite (Se^+4^) accumulation and decrease of selenate (Se^+6^), kohlrabi demonstrated antagonism between these elements contrary to pea with typical Se–I synergism. Se–I synergism in Indian mustard was demonstrated only in case of separate Se and I application but not in conditions of joint Se–I application [48]. Such multidirectional data indicate the necessity to conduct research on different agricultural crops under the same growing and enrichment conditions, since only such an approach can provide an adequate assessment of the specific characteristics of the plant response to joint enrichment with microelements.

### 4.5. The Role of Arbuscular Mycorrhizal Fungi (AMF) and Plant Growth-Promoting Bacteria (GPB)

The successful AMF utilization combined with the joint Se–I biofortification of chickpea [63] is one of the most promising approaches to the issue solution. Indeed, most of terrestrial plants demonstrate intensive symbiosis with AMF, capable to improve mineral nutrition (predominantly N, P, and K), accessibility to water, and stress resistance, due to enormous enhancement of root surface via fungi hyphae. Literature reports indicate that AMF may also be beneficial in microelements accumulation and enhancement of plant antioxidant status [64]. In this respect, special attention has been paid to the improvement of Se accumulation. It is supposed that the resulting phenomenon is connected with the fact that sulfate and phosphate transporters are decoded also by AMF genome [65,66,67], causing improvement of sulfur, phosphorus, and also Se accumulation, as selenates are accumulated via sulfate transporters, while selenites via phosphate transporters (Figure 1). The obtained, up-to-date results revealed several mycorrhizal fungi participating in the improvement of Se accumulation in host-plants: *Glomus claroideum, G. fasciculatum, G. intraradices, G. mosseae, G. versiform, Rhizophagus intraradices, Funneliformis mosseae, Alternaria seleniiphila, Alternaria astragali, Aspergillus leporis, Fusarium acuminatum,* and *Trichoderma harzianum* [68]. AMF can increase Se accumulation in plants either in ordinary conditions of low environmental Se or under Se supply [49]. The enhancement of Se and antioxidants content under AMF supply was recorded in asparagus [69], shallot plants [70] and *A. cepa* [49]. 

In case of joint Se–I biofortification of chickpea [64], a significant improvement of Se and I accumulation took place under conditions of joint foliar application. Furthermore, both phenolics content and TAA were also improved; interestingly, the improvement of plant antioxidant status was recorded both without and under Se and/or I supply. The antioxidant properties of I, its close relationship with Se metabolism in mammals, and the difficulties of plant joint biofortification with Se and I reveal the significance of these results as a new technological tool for producing functional food with high antioxidant, Se and I levels. 

Notably, the successful AMF utilization associated to the joint Se–I foliar biofortification of chickpea seems not to be linked only with changes in elements accumulation from soil. One of the possible explanations for this phenomenon may be connected with possible changes in plant hormonal status. Indeed, significant differences in Se accumulation described earlier in male and female forms of spinach [56] and hemp [71], as well as the positive effect of salicylic acid on Se–I accumulation [38,39], supports this hypothesis which would require further investigations to be proved.

Utilization of plant growth-promoting rhizobacteria for plant biofortification with microelements is another promising method for improving plant Se status and, in the latter respect, successful investigations in this field were carried out on wheat [72,73], lettuce [74] and Indian mustard [75,76]. Indeed, plant growth-promoting bacteria may be beneficial both for improving the Se status and enhancing the protection against environmental stress factors. No reports are available up to date on the effect of GPB on I accumulation.

## 5. Prospects of Iodine and Selenium Biofortification

From a practical point of view, the development of highly specific functional food products with remarkable concentrations of Se and I is connected to a rather small production volume. In this respect, sprouts and microgreens enriched with Se and I, and endowed with a high content of other antioxidants should be especially valuable. At present, a higher production of Se–I enriched mature plants seems to be possible either with AMF or phytohormones application, but both approaches need intensive investigations.

The rather controversial above-described reports relevant to the efficiency of plant joint Se–I biofortification open new fields of scientific discoveries. The strategy of further investigations should be based both on the state-of-the--art Se–I biofortification and on the peculiarities of separate plant enrichment with Se and I, especially the latter which is currently the mostly studied. A short list of possible directed investigations in joint Se–I biofortification of plants is reported below (Table 5). 

**Figure 2:**
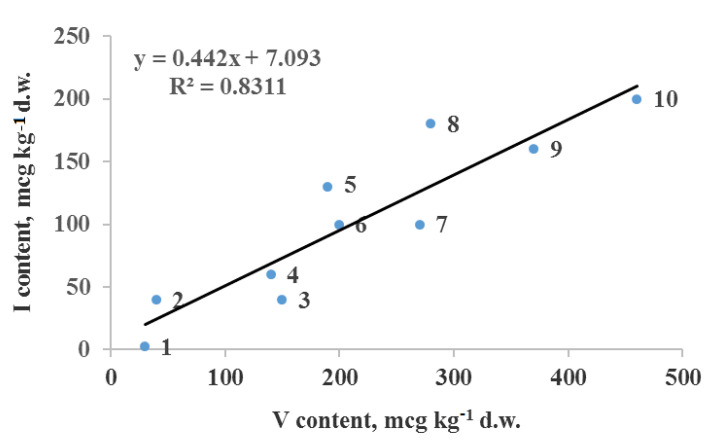
Relationship between I and V content in fruiting body of several edible mushrooms in Moscow region (Russia): *Lactarius pubescens* (1,4); *Leccinum scrabum* (3); *Paxillus involutus* (5,6,8,10); *Leccinum auranticum* (7); *Morchella esculenta* (9); (r = 0.912, *p* < 0.001, n = 10 9 (unpublished data).

The assessment of joint Se–I effect on N, P, S, Si and V relationship should be the priority (Figure 1), considering the known intensive Se effect on N, P and sulfur metabolism and participation of Si-transporters in Se accumulation. A second research target is connected with the close relationship between I and V in algae [77]. Among terrestrial plants, *Artemisia* species demonstrate a weak correlation between I and V accumulation (r = 0.624; *p* < 001; n = 14) [78]. A highly significant correlation was recorded in our research with mushrooms in Moscow region (Russia) (Figure 2).

As mushrooms belong to the plant kingdom, the relationship revealed in Figure 2 is significant considering that the interaction between I and V in plants has been poorly investigated up to date. In fact, only Smolen and coworkers [77] have tried to use this interaction for plants biofortification with I. In other investigations this relationship has not been given the appropriate importance. Taking into account the significant inter-taxa differences in I uptake [79], interesting results may be obtained in plants with high V accumulation ability, which in addition to carrot [42] and buckwheat [34], are also represented by parsley, pepper [80,81], spinach and fennel [82], radish and rocket [79]. Furthermore, it should be considered that V is also deemed a beneficial element for higher plants, participating in biosynthesis and metabolism of nitrogen compounds and improving plant growth [81,83,84]. The investigation of Grzanka et al. [77] revealed a growth stimulating effect of V and I on sweetcorn development through separate and joint I–V application, and a negative effect of joint biofortification on plant mineral composition.

Furthermore, the protection effect of Se against plant biotic and abiotic stress including heavy metals uptake should be highlighted. This approach might become especially interesting in contexts of Se–I interaction and joint biofortification. 

A further focus should be given to the effect of joint Se–I biofortification on plant antioxidant status, including accumulation of sugars which are known also to participate in plant antioxidant protection [85].

The results presented in this work indicate that a restricted number of species was used for joint Se–I biofortification, which entails the need for further determinations of the biofortification efficiency in widespread agricultural plants, such as tomato, onion, garlic, parsley, pepper, etc. Moreover, new discoveries relevant to organic Se forms and Se nanoparticles (NPs) efficiency in joint Se–I biofortification are expected. 

Finally, a special interest regards the interaction between Arbuscular Mycorrhizal Fungi (AMF) inoculation and Se–I biofortification in other agricultural crops. 

From a practical point of view the present knowledge of Se–I interaction in plants indicates the actual possibility of industrial production both of sprouts and microgreens and of several agricultural crops fortified with these elements, and emphasizes the importance of wide AMF utilization capable not only to increase plant yield and quality, but also to enhance the accumulation of Se and I.

## 6. Conclusions

Despite the attractiveness of the joint Se–I biofortification of agricultural crops, unfortunately this issue has been poorly studied and further investigations are needed for identifying the mechanism of Se–I interaction and evaluating the main factors affecting its intensity. From a general point of view, this topic may be considered as a new field of novel discoveries both in human nutrition and plant physiology. However, it is important to highlight that the current research state-of-art already shows the actual possibility to achieve the joint Se–I biofortification of several plant species at industrial scale, targeting the human health improvement. Consumers’ willingness to purchase Se–I biofortified products compared to utilization of appropriate food supplements, and Se and I levels in fortified fruits and vegetables sufficient to significantly increase Se–I consumption levels may be considered as a basis for active development of functional food production with enhanced Se and I levels.

## Figures and Tables

**Table 1:** Several examples of Se and I beneficial effects on plant growth (2019–2021 data).

Species	Dose and Method of Application	Beneficial Effect	References
Selenium			
Cucumber	1–5 µM Na_2_SeO_3_ Seedling exposure	Increase of seedling tolerance to water deficiency, by increasing the activities of the antioxidant enzymes and decrease of plasma membranes damage	[15]
Kohlrabi	Foliar supply Na_2_SeO_4_ 50–100 mg L^−1^	Increase of stem weight (by 1.35–1.61 times), yield (1.37–1.66 times), monosaccharide (1.59–2.24 times), ascorbic acid (1.54–2.01 times) and total phenolic (by 1.23–1.37 times) levels	[16]
Quinoa	2.5 and 5 mg L^−1^ soil application at early plant growth stage	Growth parameters, relative water content, photosynthetic pigments, proline, total soluble sugars, and antioxidant enzyme activities (superoxide dismutase, catalase, peroxidase, ascorbate peroxidase, glutathione reductase) increase, and decrease of malondialdehyde and H_2_O_2_ content.	[17]
Pea	10 µM Na_2_SeO_3_ or 20 µM Na_2_SeO_4_ in nutrient seedlings exposure	Protection against pea aphid *Acyrthosiphon pisum*	[18]
Tobacco	10 μM Na_2_Se0_3_ Seedling’s exposure	Increase of biomass and antioxidant capacity and reduced uptake of Cd. Higher auxin concentrations at Cd uptake compared with lack of Se supply	[19]
Sesame	Foliar supply, 5 mg L^−1^, pot experiment	Maintaining the number of leaves and increase proline accumulation, plant biomass, and grain weight per plant	[20]
Apple	Foliar 0.15 kg Se ha^−1^ (Na_2_SeO_4_)	Increase in TP, TAA and polyphenol oxidase activity	[21]
Iodine			
Strawberry	100 µM KIO_3_ bi-weekly	Enhancement of salt stress tolerance, increase in GSH and APX activity, P, K and Ca, Mn, ascorbic acid and I accumulation	[22]
Tomato	5–10 µM KI, 5-iodo salicylic acid nutrient solution	Reduction of ascorbic acid and increase of dehydroascorbic acid content in leaves. Significant increase of ascorbate peroxidase activity only with 10 µM of KI and 5-iodo salicylic acid	[23]
Potato	Soil application of KI and foliar application of KIO_3_ in doses up to 2.0 kg I ha^−1^	Increased content of I with no decrease of starch or sugar content. The highest efficiency of iodine biofortification was noted with KIO_3_ foliar spraying at 2.0 kg I ha^−1^	[24]
Apple, pear	0.5 kg KIO_3_ ha^−1^ foliar application	Increase of total soluble solids content of fruits up to 1.0 Brix	[25]

Abbreviations: TAA: total antioxidant activity; TP: total polyphenols.

**Table 2:** Needed and sufficient levels of Se and I consumption in humans and the appropriate ratios between the two elements [26,27].

	Daily Se Requirement (µg)	Daily I Requirement (µg)
Infants	10–15	40–80
Children (1–10 years)	15–30	100–140
Adolescents	45–70	180–200
Adults	60–70	200
Pregnant women	60	230
Breastfeeding women	75	260
Upper limit	300	600

**Table 3:** Examples of joint selenium and iodine biofortification of agricultural crops.

Object	Chemical Forms of Elements	Doses	Results (Se and I Content)	Se–I Interaction	Ref.
**Foliar application of Zn, I, Se, Fe Cocktail**					
Wheat 10 cultivars	ZnSO_4_+ KIO_3_^+^ Na_2_SeO_4_+Fe, EDTA	0.05% KIO_3_ 0.001% Na_2_SeO_4_	No significant effect on grain yield (338 μg Se kg^−1^; 249 μg I kg^−1^)	Decreased I levels via cocktail supply compared to single I application	[29]
Rice 7 cultivars	ZnSO_4_ KIO_3_ Na_2_SeO_4_ FeEDTA	0.05% KIO_3_ 0.001% Na_2_SeO_4_ (5 countries)	No effect on grain yield (90-584 μg Se kg^−1^; 101-335 μg I kg^−1^)	No data	[30]
**Sprouts**					
Common buckwheat (microgreens)	SeO_3_^2-^ SeO_4_ ^2-^; I^−^; IO_3_^−^	10 mg Se L^−1^ 1000 mg I L^−1^	Under Se–I combined treatment, microgreens yield was 50–70% higher than with Se and I singly (Se and I reached the contents of 0.24 μg g^−1^ DW and 216 μg g^−1^ DW, respectively)	Se decreased I by 50%, and I increased Se by 50%	[34]
Pea	KI, KIO_3_ + Na_2_SeO_3_, Na_2_SeO_4_	1000 mg I L^−1^ 10 mg Se L^−1^	No effect on chlorophyll accumulation and a slight decrease of biomass (3.9–14.1 µg Se g^–1^ DW; 152–247 µg I g^–1^ DW)	No significant relationship between elements	[35]
Pumpkin	Seed soaking + foliar application in the field	10 mg Se L^−1^, 1000 mg I L^−1^	Enhanced germination, no effect on yield (0.8–2.3 μg Se g^−1^ DW; 288–323 μg I g^−1^ DW)	Synergism in sprouts; I increased seed Se accumulation	[36]
Chervil	Na_2_SeO_4_ + KIO_3_ KI^+^ (SeCys)_2_	5 µM	Growth stimulation and TAA/TP increase only for KIO_3_+ Na_2_SeO_4_ (0.89–0.90 μg Se g^−1^ DW; 0.29–0.46 μg I g^−1^ DW)	No significant relationship between elements	[37]
**Hydroponics**					
Lettuce	Na_2_SeO_4_, KIO_3_ Salicylic acid	30 mg I m^−3;^ 8.5 mg Se dm^−3^,	SeMet and sugar increase, no effect on biomass; root Р increase and Mg decrease; the effect is dose-dependent (7.8–10.4 mg Se kg^−1^ DW; about 250 mg I kg^−1^ DW)	No data	[38]
Lettuce 6 varieties	KIO_3_ Na_2_SeO_3_ Salicylic acid	5 mg I L^−1^ 0.5 mg Se L^−1^	High varietal differences (7.5-13.7 μg Se g^−1^ DW; 75.1–304.7 μg I g^−1^ DW)	No data	[39]
Spinach	KIO_3_ Na_2_SeO_4_	10 µM I 50 µM Se	I-Se transfer factor: 3.5 to 13.4 (3–13 mg Se kg^−1^ FW; 10–25 mg I kg^−1^ FW)	I did not influence Se accumulation and vice versa	[40]
Potato	KIO_3_ Na_2_SeO_3_ Salicylic acid (SA)	39.4 µM I 6.3 µM Se	I, Se, SA did not affect tubers yield; 1 mg SA L^−1^ + (I+Se) resulted in the highest I tuber content; SA did not affect Se; N, K, Na increased and Mn, Zn decreased (100 g of fresh tubers provide 444–489% RDA Se and 47–71% RDA I)	No data	[41]
**Soil application**					
Carrot	KI Na_2_SeO_4_	4 kg I ha^−1^ + 0.25 kg Se ha^−1^	Low effect of Se and I on biochemical characteristics of roots; 100 g of biofortified carrots substantially cover the RDA for I and Se	No data	[42]
Carrot	KI Na_2_SeO_4_	4 kg I ha^−1^ and 0.25 kg Se ha^−1^	Fertilization had no effect on yield (7.24 mg Se kg^−1^ DW; 1.47 mg I kg^−1^ DW) (juice)	No data	[43]
Lettuce	Na_2_SeO_3_ Na_2_SeO_4_ KI KIO_3_	2.5 kg I·ha^−1^ + 0.5 kg Se·ha^−1^	SeMet and SeCys_2_ increase; higher biofortification level for KI and Na_2_SeO_4_ (9.4–86.7 mg Se kg^−1^; 4.2–4.7 mg I kg^−1^)	Decrease of I and Se accumulation under joint application	[44]
**Foliar application**					
Chicory	KI, KIO_3_, Na_2_SeO_3_, Na_2_SeO_4_	10 mg Se salt L^−1^; 1000 mg I salt L^−1^	No effect on plant biomass (73–85 μg Se kg^−1^ DW; 75 μg I kg^−1^ DW)	I increased Se^+4^ accumulation but decreased that of Se^+6^	[45]
Pea	KI KIO_3_ Na_2_SeO_3_ Na_2_SeO_4_	1000 mg I L^−1^ (KI or KIO_3_) 10 mg Se L^−1^ (Na_2_SeO_3_ or Na_2_SeO_4_)	No growth depression (up to 0.18–0.19μg Se kg^−1^ DW; >2% RDA for I)	Se^+4^ increased I in pea leaves, roots and pods; Se^+6^ increased seed I^−^	[46]
Kohlrabi	Na_2_SeO_3_, Na_2_SeO_4_ KI KIO_3_	1 g I L^−1^, 10 mg Se L^−1^	Se increased chlorophyll and carotene content; I increased anthocyanins; (100 g of fresh tubers provide 1.38–8.5% RDA Se and 0.79–2.01% RDA I)	Se had antagonistic effects on accumulation of I in leaves.	[47]
Indian mustard	Field experiment KI Na_2_SeO_4_	50 mg Na_2_SeO_4_ L^−1^ 100 mg KI L^−1^	Al, B increased; Cd, Sr decreased; NO_3_^−^ decreased especially under joint Se–I application; 8.6 mg Se kg^−1^ DW; 2.8 mg I kg^−1^ DW	I and Se synergism under separate supply and no effect under joint Se–I application	[48]
Chickpea	Na_2_SeO_4_ KI AMF inoculation	100 mg KI L^−1^ 50 mg Na_2_SeO_4_ L^−1^	Improvement of yield; 3305 μg Se kg^−1^ DW; 15 μg I kg^−1^ DW	Increase of Se and I fortification level by AMF; Se–I synergism	[49]
Apple, pear	KIO_3_, Na_2_SeO_4_	0.5 kg KIO_3_ ha^−1^ 0.05 kg Na_2_SeO_4_ ha^−1^	51% and 75% of the biofortified I was localized in the apple and pear peel, respectively; 20–30 μg Se kg^−1^ FW, 500–600 μg I kg^−1^ FW	No effect of Se on I accumulation	[25]

Abbreviations: TAA: total antioxidant activity; TP: total polyphenols; RDA: recommended daily allowance.

**Table 4:** Selenium and iodine content, and antioxidant status of chervil seedlings under separate and joint application of selenium and iodine [37].

Treatment	Se µg kg^−1^ d.w	I µg kg^−1^ d.w.	TAA mg GAE g^−1^ d.w.	TP mg GAE g^−1^ d.w.
Control (water)	81 ± 8b	traces	14.89 ± 1.7а	8.12 ± 1.1а
Na_2_SeO_4_	850 ± 84а	traces	12.9 ± 1.4a	7.4 ± 0.6a
KI	86 ± 9a	443 ± 115a	14.5 ± 1.5a	8.2 ± 0.9a
Na_2_SeO_4_ + KI	890 ± 91a	288 ± 75a	12.9 ± 1.4a	7.3 ± 0.6a
KIO_3_	85 ± 8b	327 ± 85a	19.9 ± 2.0b	12.6 ± 1.5b
Na_2_SeO_4_ + KIO_3_	900 ± 92a	460 ± 120a	17.8 ± 1.9b	11.7 ± 1.9b

Abbreviations: TAA: total antioxidant activity; TP: total polyphenols. Within each column, values with the same letters do not differ statistically according to Duncan test at *p* < 0.05.

**Table 5:** Fields of new discoveries.

Basic Points		Promising Directions
Known Facts	References	
Close relationship of Se with N, P, S, Si accumulation	[86]	Effect of N, P, S, Si on the efficiency of Se–I biofortification
Protective role of Se against biotic and abiotic stresses including heavy metals	[65,87]	Se–I biofortification under oxidant stress
Close relationship of Se, sugar and antioxidants accumulation	[5,88]	Effect of Se–I biofortification on sugar and antioxidants accumulation
Stimulation of I accumulation by vanadium (sweetcorn)	[77]	Effect of V on the efficiency of Se–I biofortification
Separate biofortification of tomato, pepper and onion with Se and I	[11,24,48,89,90]	Joint Se–I biofortification of tomato, onion, pepper and garlic (vegetables widely used by the population)
Increase in Se accumulation by AMF and growth promoting bacteria; a single example of Se–I biofortification of chickpea under AMF supply	[63,91,92]	Efficiency of AMF and growth promoting bacteria application on joint Se–I biofortification of different agricultural crops
High efficiency of plant biofortification with organic selenium (SeCys)_2_ and Se nanoparticles	[32,56,70,93]	Efficiency of (SeCys)_2_ and Se NP utilization in joint Se–I biofortification of plants
Division of plants to hyperaccumulators, indicators and non-accumulators of Se; with low and high iodine accumulation capacity	[7,79,94]	Efficiency of joint Se–I biofortification of hyperaccumulators, for Se hyperaccumulators and I accumulators in particular

## Data Availability

Not applicable.
